# Deprescribing in Older Adults: Is Evidence for Continued Medication Use Generalizable Beyond Age 75?

**DOI:** 10.1093/geroni/igaa057.2912

**Published:** 2020-12-16

**Authors:** Joshua Niznik

**Affiliations:** University of North Carolina at Chapel Hill, Chapel Hill, North Carolina, United States

## Abstract

Older adults over the age of 75 are severely underrepresented in many of the clinical trials used to justify the continued use of medications for chronic disease prevention in advanced age. The gaps in evidence in this population have fueled an interest in research to better understand the potential benefits and harms associated with the continued use of medications with uncertain benefit in advanced age. Deprescribing, the intentional reduction or discontinuation of medications, has recently gained traction as an important component of the prescribing process, but raises questions about the safety of stopping medications. This presentation will provide an overview of the evolution of deprescribing research and how this has shaped my career as a geriatric health services researcher. Specifically, I will address early studies that defined the field, challenges and opportunities for studying deprescribing in older adults, and future directions and priorities in deprescribing research.

